# Comparative Efficacy of Negative Pressure Wound Therapy and Conventional Treatments in the Management of Diabetic Foot Ulcers: A Systematic Review and Meta-Analysis

**DOI:** 10.3390/jcm14228051

**Published:** 2025-11-13

**Authors:** Celia Villalba-Aguilar, José Alberto Laredo-Aguilera, Lucía Villalba-Aguilar, Matilde Isabel Castillo-Hermoso, Ángel López-Fernández-Roldán, Juan Manuel Carmona-Torres

**Affiliations:** 1 Hospital Laboral Solimat, 45004 Toledo, Spain; celia.villalbaa@hotmail.com; 2 Facultad de Fisioterapia y Enfermería, Universidad de Castilla-La Mancha, 45071 Toledo, Spain; matildei.castillo@uclm.es (M.I.C.-H.); angel.lopezfernandez@uclm.es (Á.L.-F.-R.); juanmanuel.carmona@uclm.es (J.M.C.-T.); 3 Grupo de Investigación Multidisciplinar en Cuidados (IMCU), Universidad de Castilla-La Mancha, 45071 Toledo, Spain; 4 Instituto de Investigación Sanitaria de Castilla-La Mancha (IDISCAM), 45004 Toledo, Spain; 5 Facultad de Ciencias de la Salud, Universidad Rey Juan Carlos, 28922 Alcorcón, Spain; luciavillalbaaguilar12@gmail.com; 6 Hospital Nacional de Parapléjicos, 45004 Toledo, Spain; 7 Hospital Universitario de Toledo, 45004 Toledo, Spain

**Keywords:** diabetic foot ulcer, negative pressure therapy, wound healing, granulation tissue, clinical efficacy

## Abstract

**Background:** Diabetic foot syndrome is a common complication of diabetes mellitus, and its incidence is increasing due to increasing rates of overweight and an aging population. Negative pressure wound therapy has been shown to improve wound healing. This study aimed to analyze the efficacy of this therapy compared with conventional treatments in patients with diabetic foot ulcers. **Methods:** A systematic search was conducted in the following databases: PubMed, SCOPUS, CINAHL, and the Cochrane Library, and the methodological quality was assessed using the Rob2 scale. This meta-analysis was performed following the Preferred Reporting Items for Systematic Reviews and Meta-Analyses guidelines. **Results:** A total of 11 clinical trials involving 1117 subjects. The primary outcome was efficacy, which was measured by the complete healing rate, wound size, time to granulation tissue formation, adverse effects, amputations, hospital stay, and cost. This study demonstrated that this therapy improves healing (OR= −10.39, 95% CI [−14.22, −6.57]) and reduces wound size (OR = −4.11, 95% CI [−7.83, −0.39]) while potentially lowering overall costs. **Conclusions:** Limitations include heterogeneity and different variables measured in studies. Additionally, there were differences among the trials due to the lack of blinding. Although no significant differences were found in amputations or infections, this therapy reduces pain and decreases the use of antibiotics and analgesics. Its use requires individual and expert assessment to maximize its benefits.

## 1. Introduction

Diabetic foot syndrome (DFS) is a common complication of diabetes mellitus (DM) that has increasingly become a public health concern due to the increasing incidence of this disease. The prevalence of overweight and/or obesity, along with certain demographic changes, suggests that the number of adults living with DM could reach 592 million by 2035, a 55% increase from 2013 estimates [1,2,3]. This condition can be attributed to factors such as inadequate blood glucose management, distal arterial diseases, poorly perfused feet, or pressure-bearing areas on the foot [4,5]. Diabetic foot ulcers (DFUs) affect 5–25% of DM patients during their lifetime and are responsible for 85% of lower limb amputations [1,2]. The mortality rate following the development of a DFU is 2.5 times higher in patients with DM than in those without DM [2,3]. Individuals with DM exhibit impairments in inflammation, angiogenesis, tissue remodeling, and oxygenation processes, delaying the proliferation phase and hindering wound healing [2].

Wound care, including the management of DFUs, is a multidisciplinary intervention; however, it is generally the responsibility of nursing professionals [6]. This is because these professionals are capable of leading a multidisciplinary team. In Spain, there are more and more advanced practice nurses who have autonomy, greater control, and new responsibilities, especially in primary care. This is due to the epidemiological change and the high demand for care resulting from the change in the needs of the population. These changes include guaranteeing the well-being of the patient by avoiding or alleviating external forces (pressure, shear, or humidity), taking into account nutritional support, the healing of these wounds, and the prevention of infections [7], thus promoting the learning of evidence-based practice by other professionals [8,9,10].

Conventional ulcer treatment includes various alternatives, such as traditional dressings, surgical or enzymatic debridement, oxygen therapy, hydrotherapy, compression therapies, microcurrent-based therapies, platelet-rich plasma (PRP) [11], or photodynamic therapy [12]. In addition to these treatments, the Spanish Society of Vascular Surgery recommends, among other complementary options, negative pressure therapy (NPWT) for the treatment of chronic wounds [13].

NPWT was approved by the Food and Drug Administration (FDA) in 1997 [14]. This therapy is a mechanical treatment that accelerates the healing of Grade III and IV ulcers by draining excess exudate and edema and removing barriers to cellular proliferation. At the beginning of treatment, it is usually applied continuously for the first 48 h until reaching a pressure of −125 mmHg, to achieve greater release of bacterial load and reduce edema, considering that the patient does not present excessive pain or deterioration of the surrounding tissue. As wound secretion decreases, intermittent NPWT is subsequently applied, with cycles of 5 min on and 2 min off, and the pressure is adjusted on the basis of the amount of exudate [15,16].

Nevertheless, certain absolute contraindications for NPWT have been identified, including malignant wounds (except for palliative use), untreated osteomyelitis, nonenteric or unexplored fistulas, necrotic tissue with eschar, or hypersensitivity to the therapy itself. Relative contraindications include exposed blood vessels or organs; exposed anastomotic sites; respiratory emergencies; pleural, mediastinal, or chest drains; and full-thickness burns [17,18]. In a prospective pilot study, it was observed that the DFU size decreased by up to 62% within four weeks [13].

Furthermore, regarding the applications of NPWT in other types of wounds, Lin et al. [19], in their study on burns, demonstrated that NPWT accelerated healing and reduced the infection rate, although more large-scale randomized controlled trials (RCTs) are needed to provide further evidence of its efficacy in treating such wounds.

While NPWT is considered significantly effective because it improves wound healing rates and substantially reduces wound size [20], a high percentage of DFUs fail to heal, often leading to limb amputation. Various systematic reviews and meta-analyses [21,22,23,24] have evaluated the benefits of NPWT in DFU management; however, many of these studies have limitations, such as small sample sizes, heterogeneous inclusion criteria, and lack of recent randomized controlled trials (RCTs). Given the publication of new clinical trials examining NPWT effects on healing and amputation reduction, an updated systematic review and meta-analysis is warranted to provide more comprehensive and current evidence. Therefore, the aim of this study was to analyze the efficacy of NPWT compared with standard treatment in patients with DFUs, addressing gaps in previous reviews and clarifying its potential clinical benefits.

## 2. Materials and Methods

### 2.1. Information Sources

A systematic search was conducted during the first half of 2024 in the following databases: PubMed, SCOPUS, CINAHL, and the Cochrane Library. This systematic review was carried out following the Preferred Reporting Items for Systematic Reviews and Meta-Analyses (Supplementary Material S1 PRISMA) guidelines [25]. Additionally, this meta-analysis was registered in PROSPERO with the registration number CRD42024516607.

### 2.2. Search Strategy

The search strategy employed is detailed in Table 1.

The research question was formulated in the PICO format, as shown in Table 2.

### 2.3. Selection Criteria

The inclusion and exclusion criteria used for the development of this systematic review are described in Table 3.

The decision to include only clinical trials in this systematic review and meta-analysis was made to ensure the highest possible quality of scientific evidence.

### 2.4. Study Selection and Data Extraction

Two researchers (C.V.A. and J.M.C.-T) jointly conducted searches in the aforementioned databases and compiled a list of articles. After duplicate articles were filtered out, a preliminary selection was made by reading the titles and abstracts. A second selection was then conducted by reading the full texts, and the articles were selected according to the inclusion and exclusion criteria of the meta-analysis. If the two researchers disagreed during the selection process, an independent consultation was held with a third researcher (J.A.L.-A.). Data were extracted during the full-text reading process, and the following information was gathered: study authors and publication date, country, number of participants, intervention, control group, primary outcomes (efficacy measured by the following outcomes: complete healing rate where it occurred; wound size in the articles that compared it; time to granulation tissue formation in trials where it was measured; adverse effects when they occurred; number of amputations where mentioned; mean hospital stay and cost where mentioned), conclusions, and quality.

### 2.5. Tool Used for Risk of Bias Assessment

The Cochrane Collaboration is responsible for preparing, maintaining, and promoting the dissemination of systematic reviews to support healthcare decision making. The Cochrane Collaboration recommends the risk of bias 2 (RoB2) tool for assessing the risk of bias in studies included in a systematic review [26]. This tool is structured around a fixed set of bias domains, focusing on different aspects of the design, conduct, and reporting of RCTs. The reliability of RCT results depends on the extent to which potential sources of bias are avoided; thus, six aspects of bias are examined, including the randomization process, deviations from the intended interventions, missing outcome data, measurement of the outcome, selection of the reported result, and overall bias. An algorithm proposes a judgment on the risk of bias arising from each domain of the studies on the basis of the responses to the questions. This is evaluated via a Likert scale with three options (according to the criterion): “uncertain”, “low risk”, and “high risk” [27]. This tool was used by researcher C.V.A., with review by researcher J.M.C.-T.

### 2.6. Statistical Analysis

A narrative synthesis of the related studies was performed. Data on the complete healing rate where it occurred; wound size in the articles that compared it; time to granulation tissue formation in trials where it was measured; adverse effects when they occurred; number of amputations where mentioned; and mean hospital stay and cost where mentioned were analyzed. The Statistical Package for Social Sciences (SPSS) version 28, licensed by the University of Castilla-La Mancha (UCLM), was used for this analysis.

For quantitative analysis, a meta-analysis was conducted using the weighted mean differences pre- and postintervention and their respective 95% confidence intervals (CIs) for quantitative variables and odds ratios (ORs) and their 95% CIs for binary variables. Statistical data were represented via a forest plot with a significance level of α = 0.05. Statistical heterogeneity was evaluated via the I^2^ statistic: I^2^ ≤ 25%, 26–50%, or ≥51% was used to define statistically significant heterogeneity as low, moderate, or high, respectively. Finally, effect sizes from all included studies were combined to estimate an overall summary effect size with a 95% confidence interval (CI). If heterogeneity was present among studies, a random-effects model analysis was used; otherwise, a fixed-effects model analysis was employed. Statistical significance was set at 0.05. Publication bias was checked via visual inspection of the funnel plot for each meta-analysis conducted. Analyses were performed via Cochrane Collaboration’s RevMan software, version 5.4.

## 3. Results

### 3.1. Characteristics of the Included Studies

**Figure 1:**
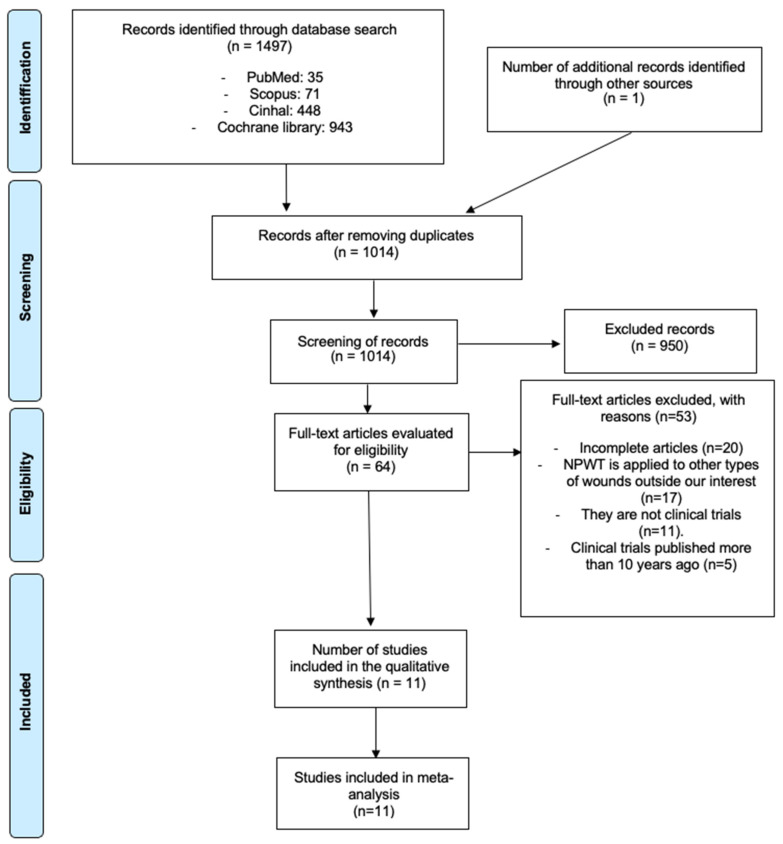
PRISMA flow diagram.

As shown in Figure 1, an initial analysis of 1497 articles sourced from the previously mentioned databases was conducted. A total of 484 duplicate articles were subsequently removed via the Mendeley reference manager. Screening was then performed first by title, resulting in the exclusion of 950 articles, followed by full-text screening, leaving 64 articles, 53 of which were excluded for the reasons explained in Figure 1, ultimately including 11 articles.

Therefore, 11 articles (Table 4) were deemed suitable for inclusion in the research, with a total sample size of 1117 participants. All participants had DM, with 547 patients in the intervention group (IG) and 570 in the control group (CG).

Table 4 shows the main characteristics of the 11 studies included in the review, showing that they were conducted in different countries (one in Germany, four in India, two in Pakistan, one in Iran, and three in Egypt). These studies also demonstrate the variation in intervention duration and follow-up periods, which contributes to confirming the efficacy of the NPWT across different populations and settings.

The number of participants in each study ranged from 40–345. The duration of the interventions ranged from 8 to 90 days. The age range of the participants was from 36.74 to 71.80 years. All participants had DM (types 1 and 2). The main characteristics of the participants included having a DFU for at least 2 weeks, a Wagner scale score of 1–4, and ulcer sizes ranging from 5 to 20 cm^2^. Additionally, most patients had hypertension (HTN), neuropathies, or ischemic heart disease.

This therapy was applied to both infected [34] and noninfected DFUs [28,29,30,31,32,33,35,36,37,38], as well as to those DFUs that had already undergone surgical intervention [28,36]. The control group received conventional treatments, including standard dressings, moist dressings, saline dressings, alginates, and silver sulfadiazine ointments.

Table 5 summarizes the use of the NPWT in the different studies. All studies mentioning NPWT treatment used VAC, and it was applied continuously, intermittently, or both; depending on the wound type, the wall suction pump could be configured to provide different levels of negative pressures (−75 to −250 mmHg). For the interface of VAC, most articles used open-pore polyurethane ether foam sponge sealed with a transparent polyurethane adhesive drape, and the conventional dressing were saline sterile gauze or silver sulfadiazine ointment dressing with roller bandages.

### 3.2. Risk of Bias Assessment

**Figure 2:**
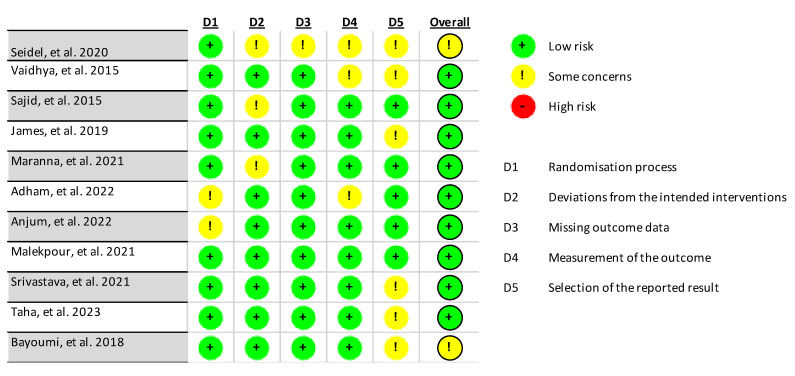
Assessment of the risk of bias of the studies included in the review [28,29,30,31,32,33,34,35,36,37,38].

**Figure 3:**
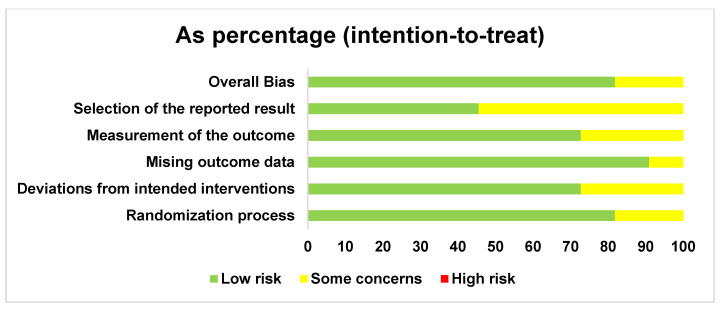
General risk of bias of studies.

In accordance with the RoB2 tool for systematic reviews of interventions, the risk of bias was assessed for the 11 included studies. These are presented in Figure 2 and Figure 3. Importantly, eight of the included studies were rated as having a low overall risk of bias, except for the studies by Seidel et al. [33] and Bayoumi et al. [37], which were rated as having an uncertain risk because they did not specify whether allocation concealment was performed.

### 3.3. Healing Rate

Among the 11 articles included in the review, 5 address the healing rate [28,30,32,33]. These studies demonstrated a better healing rate in the IG with NPWT than in the CG receiving conventional treatments. Notably, Taha et al. [28] and Anjum et al. [32] reported a 100% healing rate among patients in the IG.

**Figure 4:**
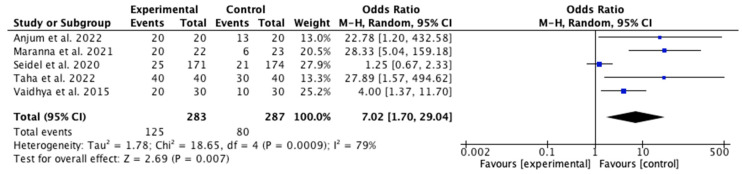
Forest plot of the wound healing rate [28,30,31,32,33].

**Figure 5:**
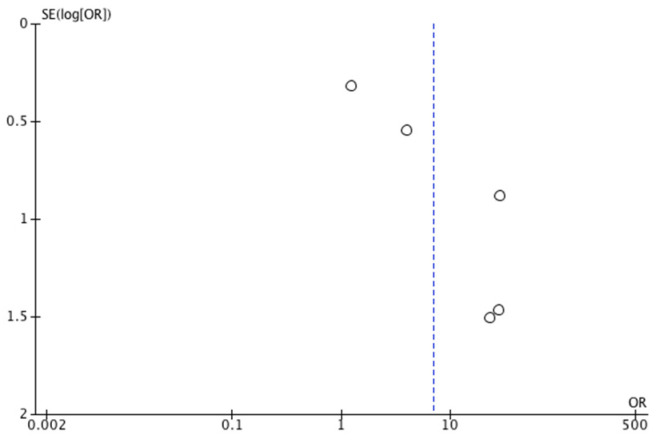
Funnel plot of the wound healing rate.

The results of the meta-analysis are depicted in Figure 4. A beneficial effect on the healing rate was observed in the IG that utilized NPWT, with an odds ratio (OR) of 7.02 (95% confidence interval [CI] 1.70, −29.04). Owing to the high heterogeneity among studies (I^2^ = 79%), a random effects model was employed. The risk of publication bias was assessed through visual inspection of the funnel plot (Figure 5), which revealed asymmetry and, therefore, a potential risk of publication bias.

### 3.4. Time to Achieve 100% Granulation Tissue Formation

Among the 11 articles included in the review, 7 [28,30,31,32,34,35,36] demonstrated a faster achievement of 100% granulation tissue formation in the IG, surpassing the CG in all studies. This was observed in both non-amputated and amputated pressure ulcers. Additionally, a study by Adham et al. [36] revealed that NPWT significantly reduces the time required to complete wound healing by enhancing granulation tissue formation during postoperative wound healing.

**Figure 6:**
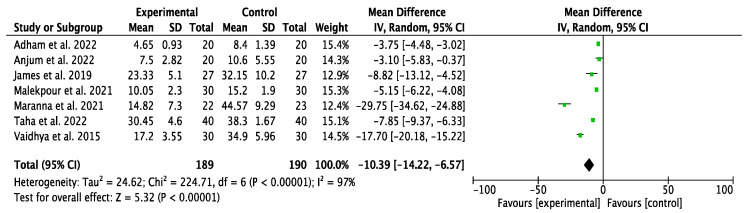
Forest plot of 100% granulation tissue time [28,30,32,34,35,36].

**Figure 7:**
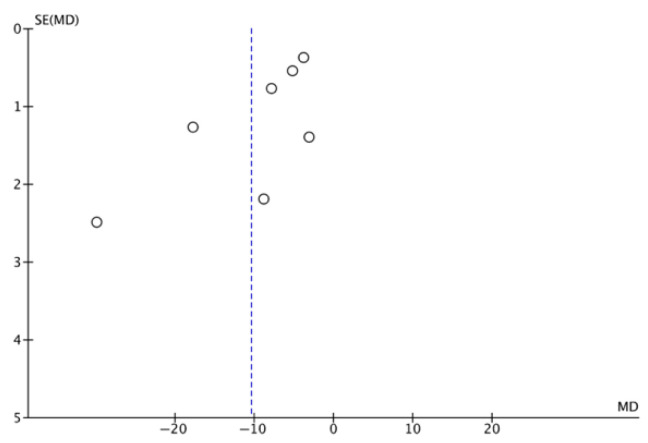
Funnel plot of 100% granulation tissue time.

The results of the meta-analysis are shown in Figure 6. A beneficial effect on the time to achieve 100% granulation tissue formation was observed in the IG using NPWT, with an odds ratio (OR) of −10.39 (95% confidence interval [CI] of −14.22, −6.57). Owing to the high heterogeneity among studies (I^2^ = 97%), a random effects model was used. The risk of publication bias was assessed through visual inspection of the funnel plot (Figure 7), which indicates that these studies are of good methodological quality, as they are located at the top of the funnel.

### 3.5. Reduction in Pressure Ulcer Size

Among the 11 articles included in this review, 7 studies [28,29,30,34,35,37,38] reported a reduction in the size of pressure ulcers. Notably, a study by Malekpour et al. [34] revealed that, compared with conventional dressings, NPWT significantly reduced the ulcer surface area (*p* = 0.008), depth (*p* = 0.002), and size (*p* = 0.02). According to the study by Taha et al. [28], the wound surface area significantly decreased in the IG after surgery for pressure ulcers compared with conventional dressings. Additionally, this study demonstrated that wound drainage ceased in 100% of patients in the IG, whereas it ceased in 65% of patients in the CG.

**Figure 8:**
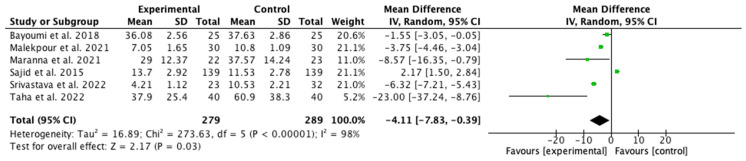
Forest plot of size reduction [28,29,30,34,37,38].

**Figure 9:**
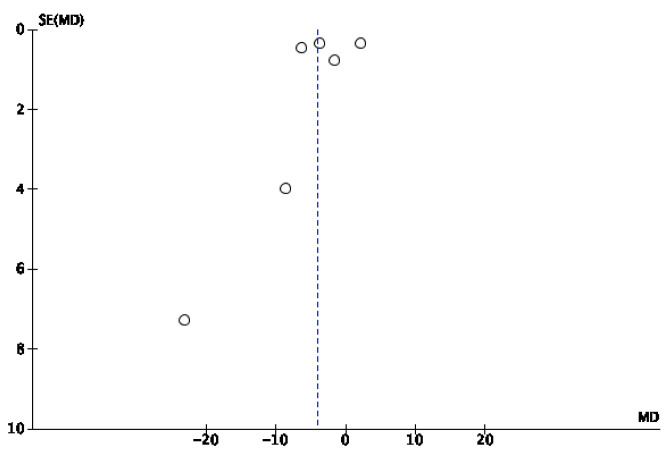
Funnel plot of size reduction.

The results of the meta-analysis are illustrated in Figure 8. A greater reduction in pressure ulcer size was observed in the IG using NPWT, with an odds ratio (OR) of −4.11 (95% confidence interval [CI] −7.83 to −0.39). Owing to the high heterogeneity among studies (I^2^ = 98%), a random effects model was applied. The risk of publication bias was assessed through visual inspection of the funnel plot (Figure 9), which revealed that the four articles at the top of the funnel exhibited similar effects, as indicated by their narrow confidence intervals.

### 3.6. Adverse Effects

**Figure 10:**
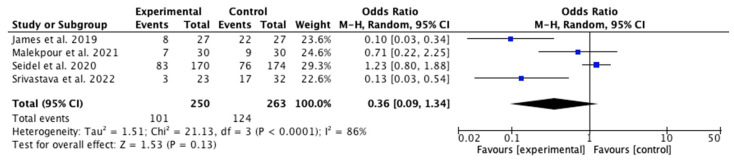
Forest plot of infections [29,33,34,35].

Among the 11 articles, 4 [28,33,34,35] compared the potential adverse effects between the IG and the CG. Although some individual studies reported differences in adverse events (fewer infections, faster bacterial load reduction, fewer hemorrhages, and lower osteomyelitis rates in the NPWT group [28,34]) the overall metanalysis did not show statistically significant differences between the two groups, as shown in the Figure 10, with a *p* value of 0.13, an odds ratio (OR) of 0.36, and a 95% confidence interval (CI) of 0.09 to −1.34. Owing to the high heterogeneity among studies (I^2^ = 86%), a random effects model was used. Additionally, according to the study by Seidel et al. [33], nine patients in the IG died, whereas six in the CG died.

**Figure 11:**
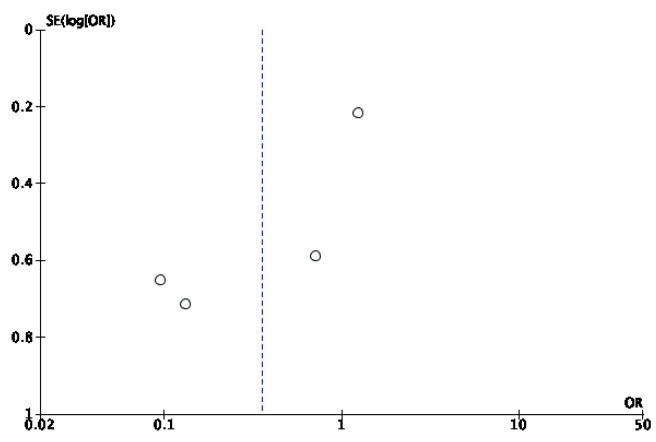
Funnel plot of infections.

The risk of publication bias was assessed through visual inspection of the funnel plot (Figure 11).

**Figure 12:**
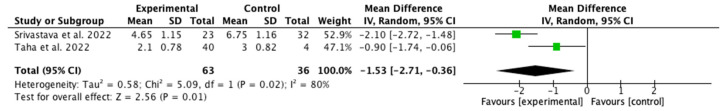
Forest plot of the pain score [28,29].

**Figure 13:**
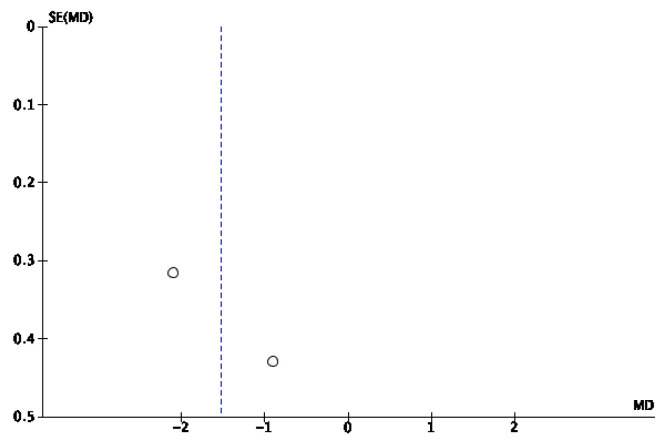
Funnel plot of the pain score.

Among the 11 articles, 2 studies [28,29] reported pain scores, with both demonstrating that patients in the IG treated with NPWT had lower scores on the visual analog scale (VAS) than patients in the CG treated with conventional treatments. The results of the meta-analysis are shown in Figure 12. A greater reduction in the VAS score was observed in the IG than in the CG, with an OR of −1.53 (95% CI −2.71, −0.36) and with a *p* value of 0.01, which demonstrated statistically significant differences. Owing to the high heterogeneity among studies (I^2^ = 80%), a random effects model was applied. The risk of publication bias was assessed through visual inspection of the funnel plot (Figure 13), where some symmetry between the studies was observed, suggesting no publication bias.

### 3.7. Amputation Rate

Minor amputations included toe amputation, toe with metatarsal bone amputation, transmetatarsal amputation (amputation of the forefoot at the mid-metatarsal level), Lisfranc disarticulation, and Pirogoff and Syme amputations.

**Figure 14:**
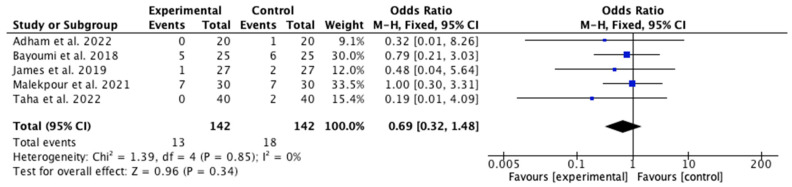
Forest plot of the minor amputation rate [28,34,35,36,37].

**Figure 15:**
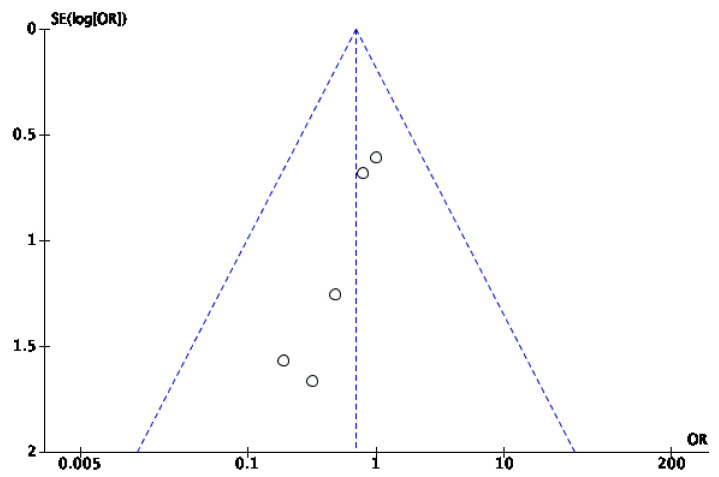
Funnel plot of the minor amputation rate.

Among the 11 articles, 5 [28,34,35,36,37] compared the number of amputations between the IG and the CG. All studies, except Malekpour et al. [34], where minor amputations were equal in both groups (7 out of 30 patients), concluded that fewer minor amputations were observed in the IG than in the CG. Notably, in the same study, 23% of both groups underwent minor amputations, but no patient in the IG required a major amputation, whereas five patients in the CG did. The meta-analysis results (Figure 14) indicate that there were no statistically significant differences between the two groups regarding minor amputations (*p* = 0.13), with an odds ratio (OR) of 0.69 (95% confidence interval [CI] 0.32 to −1.48), suggesting that the observed difference could be due to chance. As there was no heterogeneity among the included studies (I^2^ = 0%), a fixed-effects model was appropriate. The risk of publication bias was assessed through visual inspection of the funnel plot (Figure 15).

### 3.8. Hospital Stay

The analysis of hospital stay was performed in 2 of the 11 articles [30,37]. Although Vaidhya et al. [31] observed that the average cost per session was INR 500 for NPWT and INR 200 for conventional dressing, the total average treatment cost was lower for NPWT (INR 3750) compared to conventional dressing (INR 7000). When accounting for daily treatment, hospital stay, and morbidity, the cost of conventional dressing increased significantly. NPWT also required fewer analgesics and antibiotics, and patient compliance was higher.

**Figure 16:**
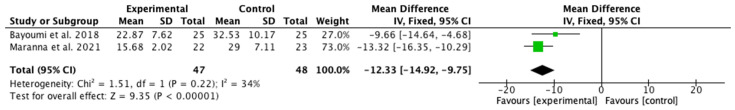
Forest plot of hospital stay [30,37].

**Figure 17:**
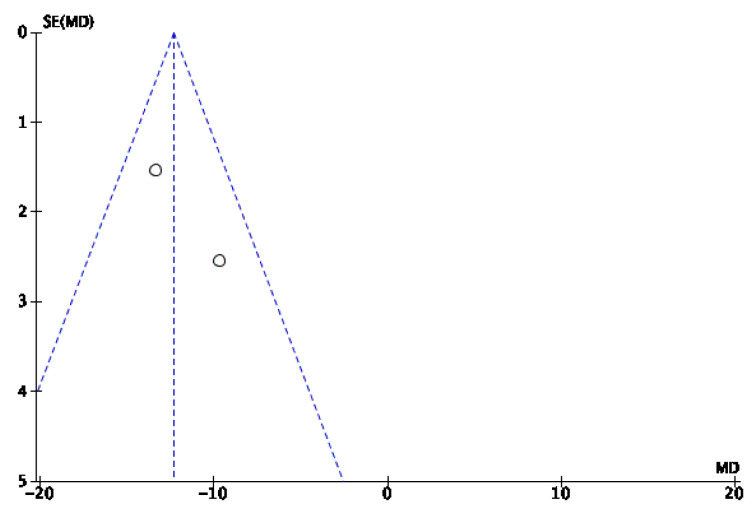
Funnel plot of hospital stay.

In the study by Maranna et al. [30], a shorter hospital stay was associated with 100% granulation tissue with NPWT in the IG than in the CG. Similarly, the study by Bayoumi et al. [37] reported a shorter hospital stay in the IG than in the CG. Nevertheless, the mean total cost of NPWT was greater (2275 ± 154 EP) than that of conventional dressings used in the CG (1976 ± 123 EP); this difference was statistically significant. Additionally, according to Srivastava et al. [29], the average cost of the materials used was greater in the IG (25,740 ± 4500 rupees) vs. (22,560 ± 1320 rupees) in the CG, but since daily bandage changes, nursing, and hospitalization costs are quite low (INR 1000 per patient), the overall cost was lower in this group than in the CG. The results of the meta-analysis are shown in Figure 16. A shorter hospital stay was observed in the IG than in the CG, with an odds ratio (OR) of −12.33 (95% confidence interval [CI] of −14.92 to −9.75). Owing to the low heterogeneity among studies (I^2^ = 32%), a fixed-effects model was applied. The risk of publication bias was assessed through visual inspection of the funnel plot (Figure 17).

## 4. Discussion

The objective of this review was to assess the efficacy of NPWT in pressure ulcers compared with that of conventional dressings in patients with DM.

Our results suggest that NPWT facilitates faster granulation tissue formation in DFUs. In the studies included in this review, NPWT was applied continuously or intermittently with negative pressure ranging from −75 to −250 mmHg to promote wound healing and drain excess exudate, as it improves the wound microenvironment and even controls infections [39]. The mechanism by which negative pressure functions in such wounds is explained in articles such as that of Argenta et al. [40], who proposed that this treatment removes excess interstitial fluid, increases angiogenesis, decreases bacterial colonization, and enhances granulation tissue formation in response to the mechanical forces exerted by the negative pressure transmitted through the sponge. Additionally, NPWT can be used as an adjunct before or after surgery, or as an alternative to surgery in palliative patients [41].

In terms of wound healing rates, the results revealed a better healing rate in the IG treated with NPWT than in the CG with conventional treatment [28,30,31,32,33]. Moreover, 100% granulation tissue was achieved in a high percentage of patients [26,27,28,29,30,31,32,33], resulting in faster wound closure due to macro-deformation, wound environment stabilization and decrease in edema, micro-deformation leading to increased cellular proliferation and angiogenesis, and decreased bacterial load, all of which led to enhanced granulation cover compared to conventional dressings [42]. However, in the study by Braakenburg et al. [43], where NPWT was used for chronic wounds, no significant difference in wound healing time between NPWT and conventional dressings was found. Nevertheless, clinical signs of inflammation and suspected infection will affect the healing rate, being lower in the IG compared to the CG according to Seidel et al. [33]. On the other hand, in the study by Braakenburg et al. [43], in which NPWT was used in chronic wounds, no significant difference was found in wound healing time between IG and the dressings used in the CG. Two years later, Blume et al. [44] reported that the DFU healing time was 1.52 times faster in the IG with NPWT than in the CG, which aligns with findings from the trials included in our review [28,29,30,34,35,36,37].

Eginton et al. [45] demonstrated that, within two weeks, DFUs were reduced by 59% in volume with NPWT versus 0% with conventional dressings. Nevertheless, a systematic review concluded that the method of measuring and evaluating ulcer size reduction and complete wound closure might affect the reliability of the results. Therefore, it is crucial to implement blinding for those measuring wound size [46].

Regarding infection rates, while some literature suggests that NPWT can enhance bacterial clearance [47,48,49], our meta-analysis did not demonstrate statistically significant differences in infection rates between NPWT and conventional dressings (*p* ≥ 0.05). These findings suggest that NPWT may not consistently reduce infection risk and should not be assumed as a guaranteed infection control measure.

According to James et al. [35], pain caused by NPWT is due to the negative suction itself, and pain was statistically lower in the IG because conventional wound treatments involve dressings that may damage new granulation when changed, thereby delaying healing and causing more pain by adhering to the wound [35,50]. Previous studies, such as those by Bechert et al. [51] and Serena et al. [52], have shown that proper pain management can enhance patient quality of life and promote better healing of chronic wounds.

Conversely, in the study by Seidel et al. [33], nine patients in the IG died, whereas six in the CG died. This suggests that NPWT is safe [53].

With respect to amputation rates, our review and meta-analysis revealed no statistically significant differences in minor amputation between NPWT and conventional dressings [28,34,35,36,37]. NPWT offers numerous benefits, as demonstrated by Chan et al. [54], who reported that, compared with conventional dressings such as iodine-based or chlorhexidine dressings, NPWT reduces the risk of complications in wounds after major lower limb amputation. While some studies reported potential reductions in minor amputation [44], evidence regarding major amputations remains limited [55,56].

The guidelines published by Everett et al. [11] for preventing limb amputation emphasize the importance of properly assessing peripheral arterial disease, adequately recognizing infections, and using antibiotics for DFU infections. Additionally, optimizing blood glucose levels is crucial for improving wound healing [2].

In terms of hospital stay, Taha et al. [28] report that patients in both groups were discharged after the second dressing change to continue receiving treatment at home, supervised by home nurses. In the study by Seidel et al. [33], treatment was started both in inpatient and outpatient care, and could continue with outpatient treatment whenever possible, even though visits needed to be performed at weeks 1, 3, 5, 12 and 16. All others performed follows-ups while patients were hospitalized, as highlighted in the study by Maranna et al. [30], who revealed statistically significant differences (*p* = 0.001), as IG patients required fewer days of hospitalization than did CG patients, which is consistent with the study by Bayoumi et al. [37]. Rai et al. [57] reported that the average hospital stay duration for patients with chronic foot wounds was 28.25 days with NPWT versus 39.17 days with saline dressings. Johari et al. [58] also reported that VAC reduced the need for hospitalization and the length of stay due to the possibility of outpatient treatment.

Patients treated with NPWT were more satisfied than those treated with conventional dressings [29,47]. This is explained by the findings of Srivastava et al. [29], who reported that the absence of daily dressing changes made IG patients feel less uncomfortable and improved their quality of life. Other trials not included in this review, conducted by Jeffcoate et al. [59] and Dsouza et al. [60], align with these findings. Likewise, Ibáñez et al. [61], in their study, demonstrated that NPWT can be applied on an outpatient basis at the patient’s home, enhancing their self-care and health empowerment, thereby improving their quality of life.

Mouës et al. [48] demonstrated that VAC therapy costs were similar to those of conventional dressings. However, Srivastava V et al. [29] and Bayoumi et al. [37] concluded that hospital costs were lower in the IG than in the CG. Similarly, Vaidhya et al. [31] reported that the average total cost of dressings for satisfactory healing in the IG was lower than that in the CG, with hospitalization costs also lower in the IG (USD 2248.59) than in the CG (USD 3102.59). Apelqvist et al. [62] also reported a beneficial direct economic cost in the use of VAC compared with conventional dressings.

Trial-based economic evaluations in certain populations (e.g., severe open fractures or routine closed incisions) have sometimes shown higher total costs with only marginal quality-adjusted life-years (QALY) gains, suggesting a low probability of being cost-effective under conventional willingness-to-pay thresholds [63]. Conversely, budget impact and device-specific analyses suggest that, in contexts where NPWT lowers infection rates or shortens length of stay, or when lower-cost single-use systems are chosen, NPWT may be economically justified [63,64,65]. In public systems with constrained budgets, targeted use in high-risk groups and careful procurement strategies may maximize value. In private or insured settings, reduced re-admissions and faster recovery may offset device costs more readily [66]. The relationship between the initial costs of the device and consumables and subsequent savings depends not only on the type of healthcare system (public or private) but also on the device provider or supplier [67], the purchasing model, reimbursement schemes, and hospital bed costs [65].

This review has several limitations. Many included studies had small sample sizes, short follow-up, inconsistent outcome reporting, and lack of blinding, which may affect reliability. Some studies involved outpatient care, potentially influencing adherence to protocols.

Regarding our review, we included studies published in English and Spanish, because this encompasses the vast majority of high-quality NPWT research, and we lacked resources for systematic translation of all other languages. The literature search was limited to a specific date, given the rapid evolution of NPWT devices, protocols, and clinical guidelines, which may have led to the omission of more recent publications. Publication bias cannot be fully excluded. Another relevant limitation is the substantial heterogeneity observed across the included trials (I^2^ > 90%). This variability likely reflects differences in baseline ulcer severity and size, concomitant standard-of-care measures (such as debridement, offloading, and infection control), and non-uniform NPWT parameters (pressure level, mode, and duration), as well as small sample sizes in some studies. Consequently, although the direction of the effect consistently favored NPWT, the magnitude of the benefit varied between studies. Heterogeneity was formally assessed using the I^2^ statistic; however, due to insufficient reporting of study-level characteristics and variability in outcome definitions, subgroup analyses or meta-regressions could not be performed to further explore potential sources of heterogeneity.

These limitations highlight the need for well-designed, multicenter trials with standardized outcome measures to better define the effectiveness of NPWT in diabetic foot ulcers.

Some studies involved outpatient care, which does not ensure adherence to dressing change protocols, especially in the CG, although they were supervised by home nurses [28]. However, in the study by Malekpour et al. [34], for patients treated with NPWT, all of the procedures in both groups were carried out by a single third-year surgical resident; to ascertain the accuracy of information provided in this study, data were provided independently by two other third-year surgical residents. Therefore, further high-quality clinical trials are needed to ensure the validity of the results and continue advancing in this area.

The strengths of the study include the diversity of data and methods, as 11 articles comparing NPWT with conventional dressings were included, covering both continuous and intermittent use with specific negative pressures, and providing a comprehensive and detailed analysis of different approaches in DFU treatment. Additionally, patient satisfaction should be assessed in all articles to evaluate how both treatments influence quality of life and limitations in daily activities, as patients treated with NPWT reported greater satisfaction due to reduced dressing change frequency, which improved their comfort and adherence to treatment [29,47].

The management of diabetic foot complications is carried out through a multidisciplinary approach involving endocrinologists, vascular surgeons, and wound care specialists. Both conservative and surgical strategies are used depending on ulcer grade and patient condition. Conservative management includes advanced wound dressings, infection control, and offloading techniques. NPWT is incorporated for complex or non-healing ulcers. When indicated, surgical procedures such as debridement or limited amputations are performed to remove necrotic tissue and facilitate wound healing [33,41].

In addition to clinical outcomes, the economic implications of NPWT should be considered. Future research should incorporate a basic cost analysis or budget impact assessment using locally relevant costs, such as device price, hospital bed-day, nursing time, and complication rates, to support informed decision making. The cost-effectiveness of NPWT may vary depending on the healthcare setting (public vs. private), the provider or supplier of the device, and local purchasing or reimbursement models.

## 5. Conclusions

The results of this study provide strong evidence that NPWT offers several significant clinical benefits over conventional treatments in the management of DFUs.

NPWT not only improves the healing rate and accelerates granulation tissue formation but also reduces wound size.

Although no statistically significant differences in amputations or infections were observed between the two groups, NPWT is safe and does not increase the incidence of severe complications while assisting in bacterial clearance. Additionally, as NPWT results in less pain, it reduces the need for antibiotics and analgesics. Therefore, this therapy should be considered more frequently for DFUs to avoid complications associated with other treatments.

Regarding cost-effectiveness, results are mixed. Some studies report lower hospital costs with NPWT, while others suggest that total cost may not differ substantially depending on device price and complication rates. Thus, the economic benefits of NPWT are context-dependent.

It is important to note that there is no universal consensus on the exact timing of switching from continuous to intermittent pressure NPWT. The decision should be based on an individual clinical assessment, considering factors such as the wound type, amount of exudate, patient tolerance, and response to treatment. It is essential for professionals to evaluate each wound individually to determine the most appropriate treatment.

With respect to clinical practice implications, NPWT should be used in many postoperative cases involving complex or large incisions to treat severe traumatic wounds, stabilize skin grafts and flaps to ensure better integration and healing, or manage infected wounds to promote a cleaner healing environment by removing infected exudate.

The NPWT is a user-friendly and easily transportable tool, although its application requires expert management to maximize benefits and minimize risks. Hence, ongoing monitoring by the nursing team throughout the healing process is recommended. Furthermore, greater standardization of NPWT application protocols, including pressure settings, duration, dressing change frequency, and patient selection criteria, is essential to improve comparability between studies and strengthen the evidence base for clinical decision-making.

## Figures and Tables

**Table 1:** Search strategy used for each database.

DATABASE	SEARCH STRATEGY
**PUBMED**	(“Negative-Pressure Wound Therapy”[Mesh]) AND “Diabetic Foot”[Mesh] Filters: Clinical Trial
**SCOPUS**	(TITLE-ABS-KEY (“vacuum-assisted closure”) OR TITLE-ABS-KEY (“negative-pressure wound therapy”) AND TITLE-ABS-KEY (“diabetic foot ulcer”) OR TITLE-ABS-KEY (“diabetic foot”) ANDTITLE-ABS-KEY (“clinical trial”)) AND (LIMIT-TO (DOCTYPE, “ar”))
**CINAHL**	TI (negative pressure wound therapy OR npwt OR acuum assisted closure OR wound vac OR vac therapy) AND TI (diabetic foot ulcer OR diabetic foot sore OR diabetic foot OR diabetic foot wound)
**COCHRANE LIBRARY**	“negative pressure wound therapy” in Title Abstract Keyword OR “vacuum assisted closure system” in Title Abstract Keyword OR “VAC therapy” in Title Abstract Keyword AND “diabetic foot ulcer” in Title Abstract Keyword OR “diabetic foot syndrome” in Title Abstract Keyword—in Trials

**Table 2:** Research question in the PICO format.

What is the Efficacy of NPWT Treatment in DFUs?			
Population (P)	Intervention (I)	Comparison (C)	Outcomes (O)
Adult patients with DM.	Use of negative pressure therapy.	Conventional treatment based on standard dressings (saline, moist, alginates, and silver sulfadiazine ointments).	Efficacy of negative pressure therapy measured by the reduction in DFU size, healing time, decrease in adverse effects, amputation rate, complete healing rate, and shorter hospital stay.

**Table 3:** Inclusion and exclusion criteria.

Inclusion Criteria	Exclusion Criteria
- Randomized clinical trials. - Patients with DM. - Adult population over 18 years of age. - Patients with diabetic foot ulcers. - Studies published in English or Spanish.	- Use of NPWT in other types of interventions outside the scope of this study. - Suspected or known allergies to the components of the different NPWT systems. - Pregnancy. - Malignancy in the target ulcer. - Trials conducted on patients with inadequate glycemic control (HbA1C > 8%) or poor general condition (coagulopathy, venous illness, concomitant use of corticosteroids, immunosuppressive or chemotherapy medications, Charcot’s joint, impalpable pedal pulses, and osteomyelitis or ischemic wounds). - Animal studies. - Clinical trials published more than 10 years ago.

**Table 4:** Main characteristics of the studies included in the review.

Authors and Country	Participants	Intervention	Control Group	Results	Conclusions	Risk of Bias
Taha et al., [28] (2023) Egypt	-N: 80 IG: 40; CG: 40 IG Age: 53.2 ± 8.0 CG Age: 56.9 ± 5.6	Both groups underwent surgical debridement of all infected and necrotic tissues. The IG was treated with NPWT. This therapy was changed every 72 h. The intervention lasted 8 weeks.	Conventional dressings changed every 24 h.	- The complete healing rate was higher at 100% in the IG vs. 75% in the CG. - The time to 100% granulation tissue formation was significantly shorter in the IG (*p* = 0.001). - The IG had a significantly greater reduction in DFU size than the CG (*p* < 0.0001). - Pain scores were lower in the IG vs. CG (*p* < 0.001). - The CG had 5% minor amputations vs. 0% in the IG.	NPWT has been shown to be effective and safe for DFUs as it reduces wound size, promotes faster granulation tissue formation, and leads to quicker cessation of drainage without increasing pain or bleeding compared to conventional dressings.	Low risk
Srivastava et al., [29] (2022) India	-N: 55 IG: 23; CG: 32 IG Age: 37.32 ± 6.84 CG Age: 36.74 ± 7.22	The IG was treated with NPWT. Dressings were changed every 7 days over the 21-day intervention period.	Conventional dressings changed daily.	- The complete healing rate was significantly better in the IG than in the CG (*p* = 0.025). - The time to 100% granulation tissue formation was significantly shorter in the IG (*p* = 0.029). - The IG had a significantly greater reduction in DFU size than the CG (*p* < 0.0001). - The mean pain score was lower in the IG (*p* < 0.001). - Polymicrobial infections occurred in 4.35% of the IG vs. 28.12% of the CG. Monomicrobial infections occurred in 8.7% and 25%, respectively. - The cost of hospitalization was significantly lower in the IG vs. CG (*p* = 0.001).	NPWT appears superior to conventional dressings, providing a higher quality of life due to earlier granulation tissue formation, faster size reduction, less discomfort, no daily dressing changes, less or no pain, and higher dressing cost but lower overall cost.	Low risk
Maranna et al., [30] (2021) India	-N: 55 IG: 22; CG: 23 IG Age: 50.23 ± 10.52 CG Age: 49.0 ± 10.14	All patients underwent initial complete debridement. The IG received NPWT with polyurethane foam. Foam was changed every 72 h. Wound measurements were taken on days 1 and 14. The intervention lasted 14 days.	Conventional saline dressings changed every 24 h with saline wash before reapplying the dressing.	- A total of 90.9% of the IG had a complete healing rate vs. 26.1% of the CG. - The time to granulation tissue formation was statistically significant in the IG with *p* = 0.001. - The reduction in DFU size in the IG was significantly greater than in the CG (*p* = 0.008). - Hospital stay was shorter in the IG than in the CG (*p* = 0.001).	NPWT led to earlier reduction in DFU size, greater granulation tissue formation, shorter hospital stays, and complete wound healing compared to conventional dressings.	Low risk
Vaidhya et al., [31] (2015) India	-N: 60 IG: 30; CG: 30 IG Age: 56.5 CG Age: 57.3	The IG received NPWT. Dressings were changed every 48–72 h. Patients in both groups were examined daily over the 8-day intervention period.	Conventional dressings were applied twice daily.	- A total of 90% of IG patients achieved complete healing vs. 76.66% of CG patients. - Granulation tissue formation time was statistically significantly shorter in the IG with *p* < 0.001.	The healing rate of DFUs was faster in the IG than in the CG. NPWT is more cost-effective for these patients.	Low risk
Anjum et al., [32] (2022) Pakistan	-N: 40 IG: 20; CG: 20 IG Age: 42.95 ± 9.29 CG Age: 46.30 ± 9.33	The IG received NPWT for 72 h. The intervention lasted 2 weeks.	Conventional saline dressings changed daily.	- The complete healing rate was 100% in the IG vs. 65% in the CG. - The time to 100% granulation tissue formation was significantly shorter in the IG than in the CG (*p* = 0.032).	VAC is more successful in achieving granulation tissue in DFUs compared to conventional dressings.	Low risk
Seidel et al., [33] (2020) Germany	-N: 345 IG: 171; CG: 174 IG Age: 67.8 ± 11.9 CG Age: 67.6 ± 12.3	The IG received NPWT. Both groups were evaluated at weeks 1, 3, 5, 12, and 16. The intervention lasted 16 weeks.	Conventional moist dressings.	- A total of 14.6% of IG patients achieved complete healing vs. 12.1% of CG patients. - A total of 36.8% of the IG experienced at least one adverse event vs. 33.33% of the CG. Mortality was 5.3% in the IG vs. 3.5% in the CG.	NPWT was not superior to conventional moist dressings in DFUs. Overall, the wound closure rate was low.	Some concerns
Malekpour et al., [34] (2021) Iran	-N: 60 IG: 30; CG: 30 IG Age: 70.31 ± 5.92 CG Age: 71.80 ± 6.32	The IG received NPWT changed every 48 h. Data were recorded twice weekly for 3 months. All patients were followed until complete DFU closure.	Conventional dressings changed twice daily.	- The time to 100% granulation tissue formation was significantly shorter in the IG than in the CG (p = 0.002). - The IG showed significantly greater reduction in DFU surface area and depth than the CG (p = 0.008 and p = 0.002, respectively). - A total of 30% of CG patients had osteomyelitis vs. 23% in the IG. - A total of 23% of both groups had minor amputations; however, 0% of the IG had major amputations vs. 16.5% in the CG.	Better efficacy was observed in treating infected DFUs with NPWT, leading to higher healing rates, size reduction, shorter posttreatment disability duration, and fewer major and minor amputations. However, no differences were observed between the treatments in terms of complications.	Low risk
James et al., [35] (2019) India	-N: 54 IG: 27; CG: 27 IG Age: 55.9 CG Age: 52.9	All patients underwent debridement. DFU area was assessed at the beginning and end of the study over 3 weeks. The IG received continuous NPWT, with dressings changed every 48 h.	Conventional saline dressings changed daily and evaluated every 48 h.	- The time to granulation tissue formation was statistically significant in the IG with *p* < 0.0001. - The CG had a mean pain score of 4 on the VAS vs. 3 in the IG. - There was a higher percentage of infections in the CG (81.48%) vs. the IG (29.63%). A total of 59.26% of IG patients had no nosocomial infections vs. 44.44% in the CG. - A total of 3.7% of IG patients had an amputation vs. 7.4% in the CG.	NPWT significantly reduces the time to complete healing, granulation tissue formation, and more rapidly decreases DFU area compared to conventional dressings.	Low risk
Adham et al., [36] (2022) Egypt	-N: 40 IG: 20; CG: 20 IG and CG Age: N/A	Patients first underwent surgical debridement or minor amputations before treatment. The IG received continuous NPWT. Outcomes were measured at weeks 2, 4, 6, 8, and 10. The intervention lasted 10 weeks.	Conventional saline gauze dressings.	- The time to 100% granulation tissue formation was statistically better in the IG (*p* < 0.01). - A total of 5% of CG patients had minor amputations vs. 0% in the IG.	The time to complete wound healing was significantly better in the NPWT VAC group compared to conventional dressings.	Low risk
Bayoumi A, et al. [37] (2018) Egypt	-N: 50 IG: 25; CG: 25 IG and CG Age: >60 years	All wounds were debrided. The IG was treated with NPWT. The intervention lasted 3 weeks.	Conventional moist dressings.	- The IG had a significantly greater reduction in DFU size than the CG (p < 0.05), as well as in the percentage of ulcer area (p = 0.001%). - One more patient in the CG than in the IG (5 vs. 6) required a minor amputation, although there were no significant differences between the groups (p = 0.13). - Hospital stay was shorter in the IG than in the CG (22.87 ± 7.62 days vs. 32.53 ± 10.17 days). Nevertheless, the average cost of conventional dressings was lower compared to NPWT (2275 ± 154 EGP vs. 1976 ± 123 EGP), but there were no significant differences in total cost.	VAC is more effective than conventional dressings in treating DFUs in diabetic patients. Hospitals should consider this therapy as an essential modality in the treatment of these wounds.	Some concerns
Sajid et al., [38] (2015) Pakistan	-N: 278 IG: 139; CG: 139 IG Age: 56.8 ± 11.3 CG Age: 55.9 ± 10.9	The IG received intermittent NPWT at −125 mmHg, changed every 48–72 h. Both groups were evaluated weekly for 2 weeks.	Conventional moist dressings changed daily.	- DFU size reduction was significantly greater in the IG vs. CG (*p* < 0.001).	NPWT was more effective than conventional dressings in managing DFUs.	Low risk

Abbreviations: (1) N: number of total participants; (2) IG: intervention group; (3) CG: control group; (4) NPWT: negative pressure therapy; (5) AE: adverse event; (6) DFU: diabetic foot ulcer; (7) VAS: visual analog scale; (8) N/A: not available; (9) VAC: vacuum-assisted closure; (10) EGP: Egyptian Pounds.

**Table 5:** Summary of the use of negative pressure wound therapy.

Authors	Mode	Pressure Measurements (mmHg)	Interface Layer or Foam	Treatment During the Intervention and/or Graft
Taha et al., [28] (2023)	Intermittent (5 min on, 2 min off)	−125 mmHg	- IG: Open-pore (400–600 μm) black polyurethane ether foam that is cut to fit the size and shape of the wound cavity. The foam was sealed with a transparent polyurethane adhesive drape to completely cover the foam and about 3–5 cm of the surrounding skin. - CG: Comprehensive wound wash with physiological saline and then covered with sterile gauze and nonadherent dressing and roller bandages.	N/A
Srivastava et al., [29] (2022) India	Intermittent or continuous	For 6 days, an evacuation tube embedded in foam was linked to vacuum at a negative pressure of −125 mmHg. Depending on the wound type, the wall suction pump could be configured to provide different levels of negative pressure (100–250 mmHg)	- IG: Foam-based dressing covered with an adhesive drape. - CG: Saline-moistened gauze dressings.	N/A
Maranna et al., [30] (2021) India	Continuous	−125 mmHg	- IG: Polyurethane foam of size 400–600 μm, trimmed to the appropriate size and geometry of the ulcer. The foam was covered by an adhesive tape and secured air-tight by the therapeutic regulated accurate care (TRAC) system. - CG: Normal saline-soaked gauze.	Vacuum-assisted closure VAC^®^ therapy (KCI, San Antonio, TX, USA).
Vaidhya et al., [31] (2015) India	Intermittent (30 min on and 30 min off)	−80 to −150 mmHg	- IG: Piece of foam cut according to size and shape of ulcer, and adhesive transparent dressing (OpSite by Smith & Nephews). - CG: Cleaning with povidone iodine solution with or without hydrogen peroxide, and applying moist gauze to wound and dressing closed by cotton bandage.	Kinetic Concepts Incorporated (KCI) VAC^®^ modified
Anjum et al., [32] (2022) Pakistan	Continuous	−80 to −125 mmHg	- IG: Foam-based dressing concealed within the cemented wrap to create the air-tight seal. - CG: Saline-moistened gauze dressings.	VAC
Seidel et al., [33] (2020) Germany	Intermittent and continuous	N/A	- IG: Granufoam black or silver, white foam, Renassys-F/P, and Renassys-G. - CG: Wet gauze dressings.	Kinetic Concepts Incorporated (KCI) and Smith & Nephew (S&N)
Malekpour et al., [34] (2021) Iran	Intermittent (5 min on, 2 min off)	−75 to–100 mmHg	- IG: Open-pore polyurethane ether foam sponge and a semi-occlusive adhesive cover. - CG: Silver sulfadiazine ointment dressing.	VAC (FAPSCO, Tehran, Iran)
James et al., [35] (2019) India	Continuous	−125 mmHg	- IG: Saline-soaked gauze piece after it was thoroughly cleaned. VAC was applied by placing sterile pads in two layers with a 16Fr Ryle’s tube placed between the two layers; then, the wound was sealed by a sterile transparent polyurethane sheet. - CG: Saline-soaked gauze piece and two layers of sterile gauze piece were placed on the dressing and secured with roller bandages.	VAC
Adham et al., [36] (2022) Egypt	Continuous	−125 mmHg	- IG: Foam dressing. - CG: Saline and sterile gauze.	VAC
Bayoumi et al., [37] (2018) Egypt	N/A	N/A	- IG: Double layer of polyethylene sheets was held firmly in place over the wound. - CG: Conventional moist dressings.	VAC
Sajid et al., [38] (2015) Pakistan	Intermittent	−125 mmHg	- IG: Opsite^®^ sheet, corrugated drain, and sterilized foam. - CG: Advanced moist wound therapy (AMWT).	VAC

## Data Availability

All databases employed and/or analyzed in this study are available from corresponding author upon reasonable request.

## Supplementary Materials

The following supporting information can be downloaded at: https://www.mdpi.com/article/10.3390/jcm14228051/s1, Supplementary Material S1: PRISMA 2020 checklist [68].
